# Colonic bacterial composition is sex-specific in aged CD-1 mice fed diets varying in fat quality

**DOI:** 10.1371/journal.pone.0226635

**Published:** 2019-12-18

**Authors:** Allison L. Unger, Korin Eckstrom, Thomas L. Jetton, Jana Kraft

**Affiliations:** 1 Department of Animal and Veterinary Sciences, The University of Vermont, Burlington, Vermont, United States of America; 2 Department of Microbiology and Molecular Genetics, The University of Vermont, Burlington, Vermont, United States of America; 3 Department of Medicine, Division of Endocrinology, Metabolism and Diabetes, The University of Vermont, Colchester, Vermont, United States of America; University of Illinois, UNITED STATES

## Abstract

Evidence suggests that sex influences the effect of diet on the gut bacterial composition, yet, no studies have been performed assessing dietary fatty acid composition (*i*.*e*., fat quality) in this context. This study examined the effect of dietary fat quality on colonic bacterial composition in an aged, genetically-diverse mouse population. CD-1 mice were fed isoenergetic diets consisting of (1) control fat (CO; “Western-style” fat blend), (2) CO supplemented with 30% fish oil, (3) CO supplemented with 30% dairy fat, or (4) CO supplemented with 30% echium oil. Fecal samples were collected at mid-life and aged (reproductively senescent) time points. Overall, the abundance of *Bacteroidetes* was greater in mice fed echium oil compared to mice fed the control fat. Examination of colonic bacterial relative abundance also revealed sex differences, with 73 bacterial taxa being differentially expressed in males and females. Notably, results showed a strong interactive effect among the diet, sex, and age of mice which influenced colonic bacterial relative abundance and alpha diversity. In males, supplementation of the diet with dairy fat or echium oil caused the abundance of *Bacteroidetes* and *Bacteroides* to change with age. Additionally, supplementation of the diet with fish oil induced sex-dependent changes in the alpha diversity of aged mice compared to mid-life. This work supports that sex is a critical factor in colonic bacterial composition of an aged, genetically-heterogenous population. Moreover, this study establishes that the effectiveness of dietary interventions for health maintenance and disease prevention via direct or indirect manipulation of the gut microbiota is likely dependent on an individual’s sex, age, and genetic background.

## Introduction

The gut harbors a dynamic and complex bacterial community that plays a critical role in disease prevention and pathogenesis [1–3]. Compared to the number of the host’s cells in the body, the gut bacteria comprise a comparable cell number and contribute to as much as 0.3% of an individual’s body weight [4]. Yet, the number of bacterial genes is 100-fold greater than that of the human host [5] and, consequently, they influence systemic host metabolism and immunity [3,6]. Conversely, many host-related factors, including age, diet, and genetic makeup, shape the gut bacterial community structure and hence their functional and metabolic characteristics [2,7,8].

The composition of the gut bacteria changes throughout an individual’s lifetime [9]. An infant has a highly variable gut bacterial population that rapidly develops until approximately the age of three [10]. During adulthood, the gut bacterial composition is relatively stable and dominated by the phyla *Firmicutes* and *Bacteroidetes* [11]. However, the gut bacteria population undergoes dramatic changes during the aging process. For instance, aging has been associated with a general decrease in bacterial diversity and increased prevalence of *Bacteroidetes* [12]. Changes in the gut bacterial composition have been correlated with inflammation, frailty, and several comorbidities [13,14]. While it is thought that the gut bacteria are key drivers of chronic low-grade inflammation, termed “inflammaging” [15] or immunosenescence [16], the underlying mechanisms are unknown. Yet, as the number of elderly individuals (65 years and older) is increasing worldwide [17], understanding the role of the gut bacterial community structure in response to lifestyle patterns (*e*.*g*., diet preference) in an aged population is of critical importance for health maintenance and disease prevention.

The “Western-style” diet, generally considered to be a dietary pattern high in saturated fatty acids (SFA) and simple carbohydrates while low in dietary fiber, has been shown to negatively impact the gut bacterial community structure, characterized by an increased ratio of *Firmicutes* to *Bacteroidetes* and a loss of bacterial diversity [18–22]. In addition, shifts in gut bacterial composition in response to a “Western-style” diet have been linked to metabolic risk factors such as obesity [23–27], inflammation [21,28,29], and altered energy homeostasis [22,23,29,30]. Nevertheless, the effect of dietary fatty acid (FA) composition on the gut bacterial community structure is not well established. Recent evidence in mouse studies demonstrated that diets comprising of oils rich in monounsaturated FA (MUFA, *e*.*g*., olive oil) or cold-water fatty fish rich in long-chain polyunsaturated FA (PUFA) have a range of effects on the gut bacterial composition compared to diets high in SFA [21,22,28]. However, it is important to note that these studies involved short-term diet manipulations, ranging from 4–11 weeks.

A recent review [31] highlighted a striking sexual dimorphism in mammalian phenotypes and identified both a sex-bias and/or a lack of analysis by sex in medical research. Sex has been shown to play a key role in shaping the gut bacterial composition [32–34]. Although the mechanisms are not fully understood, the relationship between sex and the gut bacterial community structure is thought to be driven by differences in circulating sex hormones [35]. Although data are accumulating indicating that diet may affect gut bacterial community structure differently in males than in females [35–38], there are only a few mouse studies examining the interaction between the gut microbiota and diet have incorporated both sexes into the experimental design.

It is also not known whether there are sex-specific physiological effects of a long-term dietary intervention varying in fat quality in an aged population. Our study sought to compare the impact of three diets with different fat quality (*i*.*e*., FA composition) to the fat quality of a “Western-style” diet on the density, abundance, and diversity of colonic bacteria in a genetically heterogeneous, aged mouse population. Specifically, we supplemented diets with one of three dietary sources of unique FA conferring a distinct FA composition to each diet: fish oil (FO), rich in eicosapentaenoic acid and docosahexaenoic acid; dairy (butter) fat (BO), rich in short-, odd-, branched-chain FA, vaccenic acid, and conjugated linoleic acids; or echium oil (EO), rich in *α*- and *γ*-linolenic acids and stearidonic acid. We hypothesized that a diet supplemented with dietary fats rich in unique FA (*i*.*e*., fish oil, dairy fat, and echium oil) lowers the ratio of *Firmicutes* to *Bacteroidetes* and enhances the diversity of colonic bacteria compared to a control fat reflecting the FA profile of a typical U.S. American diet [39] (*i*.*e*., Western-style diet) at mid-life and aged life stages. The objectives were to i) identify and quantify the composition of colonic bacteria, ii) classify the colonic bacteria at the phylum, genus, and operational taxonomic unit (OTU) level via 16S rRNA gene sequencing, and iii) analyze differences in abundance and diversity by age, diet, and sex.

## Results

### Metabolic parameters

**Fig 1:**
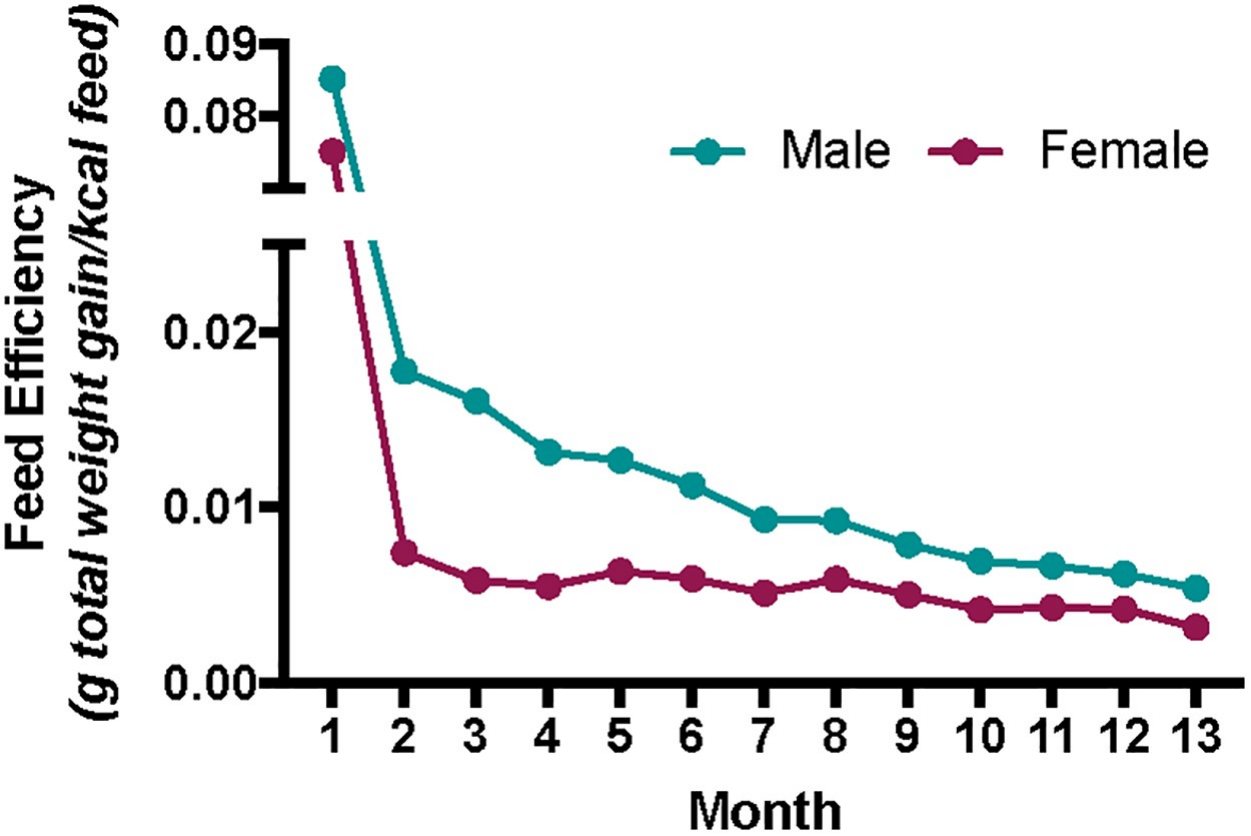
Feed efficiency (final body weight (g)—Initial body weight (g) / total feed (kcal) consumed) during the study of male and female CD-1 mice collapsed by diet group. Male CD-1 mice exhibited a higher feed efficiency compared to female CD-1 mice (*P* < 0.05).

Body weight, feed intake, and feed efficiency were not different among diet groups (S1 Table). As expected, feed efficiency declined with age in both sexes (*P* < 0.001), and males had a greater body weight (*P* < 0.001) and feed efficiency (*P* < 0.001; Fig 1) than females (Table 1). Metabolic parameters of mice for all diet groups are shown in S1 Table.

**Table 1:** Body weight, feed intake, and feed efficiency of male and female CD-1 mice collapsed by diet group and age. Values are expressed as mean ± standard error of the mean. *P < 0.05; **P < 0.01; ***P < 0.001.

Parameter	Male	SEM	Female	SEM	*P* value						
					D^1^	S^2^	A^3^	DxS	DxA	SxA	DxSxA
**Body weight (g)**	62.1	1.5	40.2	2.4	-	***	*	-	-	-	-
**Feed intake (g/day)**	3.6	0.1	3.1	0.1	-	-	**	-	**	-	-
**Feed efficiency**^4^	0.006	0.000	0.004	0.001	-	***	***	-	-	***	-

^1^D: Diet.

^2^S: Sex.

^3^A: Age.

^4^Final body weight (g)–initial body weight (g) / total feed (kcal) consumed.

### Colonic bacterial density and abundance

As obesity has been previously linked to changes in gut bacterial community structure [20,23–27,40], we sought to investigate the effect of animal weight on the colonic bacterial composition in aged mice. Surprisingly, when included as a covariate in the statistical model, body weight was found to have no effect on colonic bacterial density or abundance at the phylum level. As a covariate, body weight affected the abundance of several genera including *Allobaculum*, *Anaerostipes*, *Blautia*, and *Lachnoclostridium* (*P* < 0.05).

**Fig 2:**
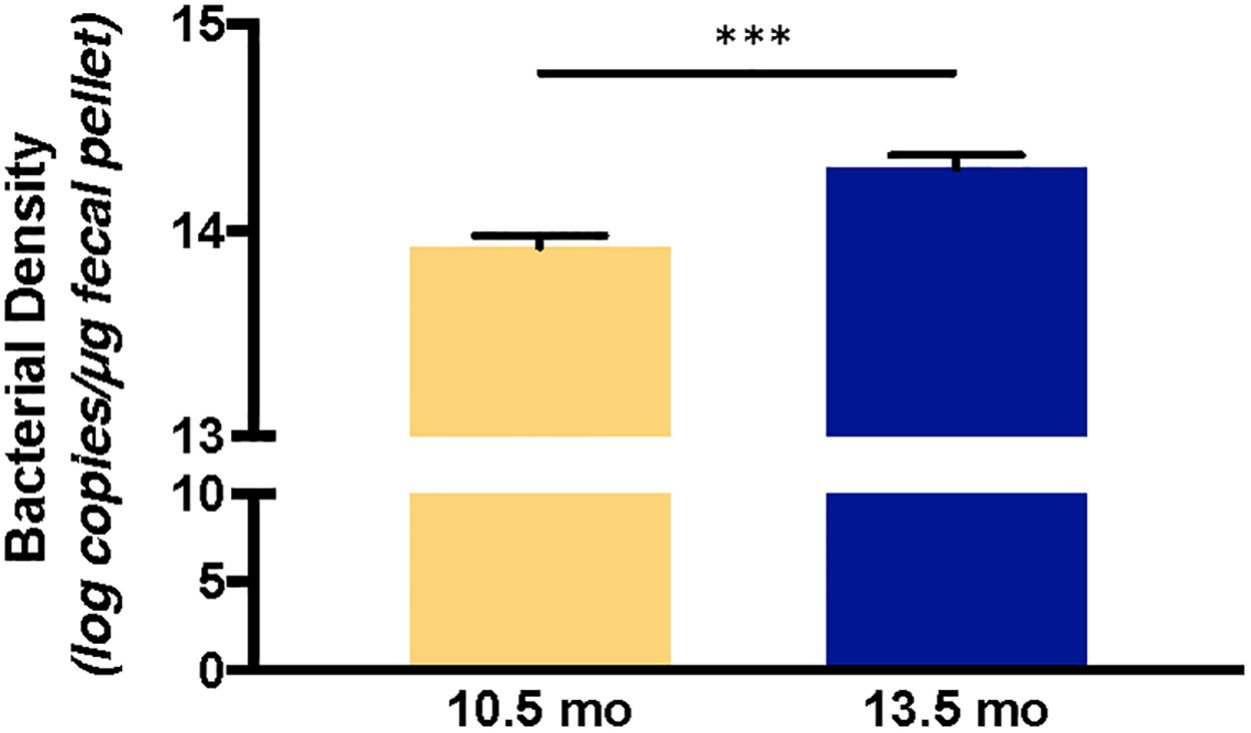
Colonic bacterial density (log copies/μg fecal pellet) of CD-1 mice at 10.5 and 13.5 months of age collapsed by diet group and sex. Values are expressed as mean ± standard error of the mean. *P < 0.001.

Age impacted the colonic bacterial density; mice at 13.5 months of age had a greater bacterial density than at 10.5 months of age (14.3 versus 13.9 log copies/μg fecal pellet, *P* < 0.001; Fig 2). Colonic bacterial density for diet groups, collapsed by sex and age, is shown in S2 Table.

**Fig 3:**
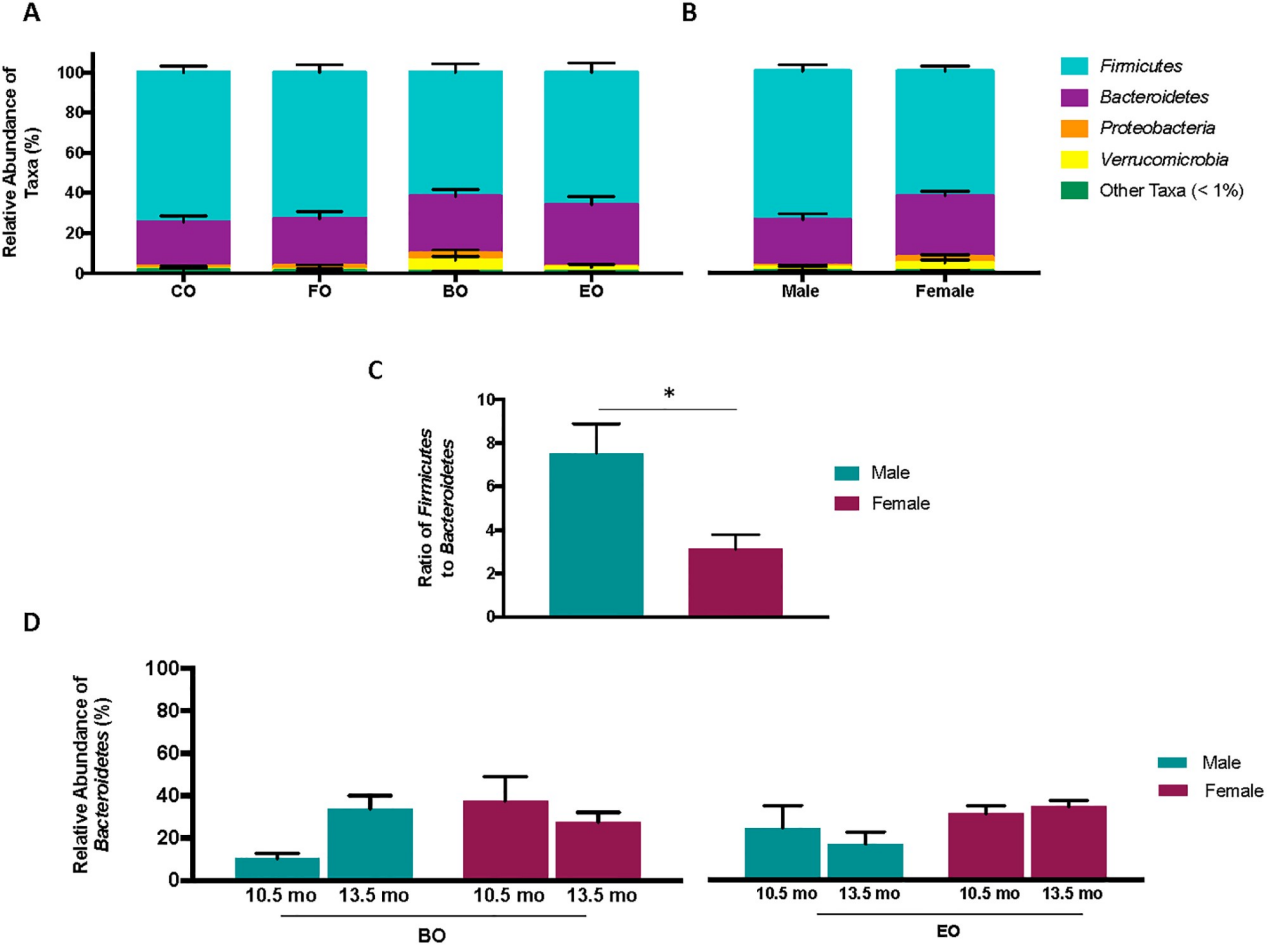
**(A) Relative abundance of colonic bacteria at the phylum level of male and female CD-1 mice collapsed by sex and age**. CO: CD-1 mice fed a “Western-style” control fat. FO: CD-1 mice fed CO supplemented with 30% fish oil. BO: CD-1 mice fed CO supplemented with 30% dairy fat. EO: CD-1 mice fed CO supplemented with 30% echium oil. Abundance by counts of *Bacteroidetes* was greater in EO-fed mice compared to all other diet groups (*P* < 0.05). **(B) Relative abundance of colonic bacteria at the phylum level of male and female CD-1 mice collapsed by diet group and age**. Abundance by counts of *Bacteroidetes* was greater in females compared to males (*P* < 0.05). **(C) Ratio of *Firmicutes* to *Bacteroidetes* of male and female CD-1 mice collapsed by diet group and age. (d) Shifts in relative abundance of the colonic *Bacteroidetes* of BO-fed male and female CD-1 mice at 10.5 and 13.5 months of age. Shifts in relative abundance of the colonic *Bacteroidetes* of EO-fed male and female CD-1 mice at 10.5 and 13.5 months of age**. In BO-fed males, abundance by counts of *Bacteroidetes* was greater at 13.5 months of age compared to 10.5 months of age (*P* < 0.01). In EO-fed males, abundance by counts of *Bacteroidetes* was lower at 13.5 months of age compared to 10.5 months of age (*P* < 0.01). Values are expressed as mean ± standard error of the mean. *P < 0.05; **P < 0.01.

At the phylum level, abundance by counts of *Bacteroidetes* was 100% greater in EO-fed mice than in CO-fed mice, respectively (S2 Table; relative abundance displayed in Fig 3A; *P* < 0.05). No differences among diet groups were observed in the abundance of other phyla. The abundance of *Bacteroidetes* (Table 2; relative abundance displayed in Fig 3B) was 17% greater and the ratio of *Firmicutes* to *Bacteroidetes* (Fig 3C) was 59% lower in females than in males (*P* < 0.05). Notably, an interaction between diet, sex, and age (Table 2; *P* < 0.001) revealed that colonic bacterial abundance of *Bacteroidetes* changed inversely as aging occurred in BO-fed males vs. BO-fed females, and in EO-fed males vs. females (*P* < 0.01; relative abundance displayed in Fig 3D). Furthermore, at 13.5 compared to 10.5 months of age, BO-fed males had a greater abundance of *Bacteroidetes* (10715 versus 3145, respectively *P* < 0.01), while EO-fed males had a lower abundance of *Bacteroidetes* (4360 versus 11954; *P* < 0.01). The abundance of colonic bacteria at the phylum level for diet groups, collapsed by sex and age, is shown in S2 Table, while the relative abundance of bacteria at the phylum level for diet groups, segregated by sex and age, is shown in S1 Fig.

**Table 2:** Colonic bacterial abundance by counts at the phylum level^1^ of male and female CD-1 mice collapsed by diet group and age. Values are expressed as mean ± standard error of the mean. *P < 0.05; ***P < 0.001.

Phylum	Male	SEM	Female	SEM	*P* value						
					D^2^	S^3^	A^4^	DxS	DxA	SxA	DxSxA
***Bacteroidetes***	6583	1115	7722	887	*	*	-	-	-	-	***
***Firmicutes***	22838	2935	15219	1565	-	-	-	-	-	*	-
***Proteobacteria***	312	82	843	452	-	-	-	-	-	-	-
***Verrucomicrobia***	590	279	614	233	-	-	-	-	-	-	-

^1^Mean relative abundance > 1%.

^2^D: Diet.

^3^S: Sex.

^4^A: Age.

At the genus level, colonic bacterial abundance did not differ among diet groups (S3 Table). *Roseburia* was over 20% more abundant in females than males (Table 3; relative abundance displayed in S2 Fig; *P* < 0.05). Compared to 10.5 months of age, abundance, by counts, of *Barnesiella* (315 versus 1406; *P* < 0.001), *Bilophila* (185 versus 515; *P* < 0.01), *Ruminococcus* (319 versus 951; *P* < 0.01), and Turicibacter (323 versus 1329; *P* < 0.01) was greater at 13.5 months of age compared to 10.5 months of age in females. The abundance by counts of *Alistipes* changed from 1468 to 237 in CO-fed males and from 331 to 1141 counts in CO-fed females (10.5 versus 13.5 months of age, respectively; relative abundance displayed in S3 Fig; *P* < 0.01). In addition, the abundance of *Bacteroides* changed from 1484 to 7314 counts in BO-fed males but from 8643 to 3108 counts in EO-fed males (10.5 versus 13.5 months of age, respectively; relative abundance displayed in S3 Fig; *P* < 0.05). The abundance of colonic bacteria at the genus level for diet groups, collapsed by sex and age, is shown in S3 Table. The relative abundance of colonic bacteria at the genus level for diet groups, segregated by sex and age, is shown in S3 Fig. These data suggest that sex is an important factor in the effect of dietary fat quality on colonic bacterial composition in aged mice.

**Table 3:** Colonic bacterial abundance by counts at the genus level^1^ of male and female CD-1 mice collapsed by diet group and age. Values are expressed as mean ± standard error of the mean. *P < 0.05; **P < 0.01; ***P < 0.001.

Genus	Male	SEM	Female	SEM	*P* value						
					D^2^	S^3^	A^4^	DxS	DxA	SxA	DxSxA
***Akkermansia***	590	279	614	233	-	-	-	-	-	-	-
***Alistipes***	799	109	1608	211	-	-	-	-	-	***	*
***Allobaculum***	3151	880	997	571	-	-	-	-	-	-	-
***Anaerostipes***	323	51	371	82	-	-	-	-	-	-	-
***Bacteroides***	4469	908	4085	698	-	-	-	-	-	-	*
***Barnesiella***	326	57	934	204	-	-	***	-	-	**	-
***Bilophila***	216	44	373	77	-	-	*	-	-	*	-
***Blautia***	448	43	553	84	-	-	-	-	-	-	-
***Clostridium***	7167	981	6325	660	-	-	-	-	-	-	-
***Eubacterium***	210	43	363	60	-	-	-	-	-	-	-
***Lachnoclostridium***	338	31	350	45	-	-	-	-	-	-	-
***Lactobacillus***	7716	1734	2372	507	-	-	-	-	-	-	-
***Oscillospira***	813	105	784	72	-	-	-	-	-	-	-
***Parabacteroides***	904	210	1023	175	-	-	-	-	-	-	-
***Roseburia***	380	55	494	58	-	*	-	-	-	-	-
***Ruminococcus***	269	53	678	320	-	-	-	-	-	*	-
***Turicibacter***	723	179	894	260	-	-	**	-	-	*	-

^1^Mean relative abundance > 1%.

^2^D: Diet.

^3^S: Sex.

^4^A: Age.

**Fig 4:**
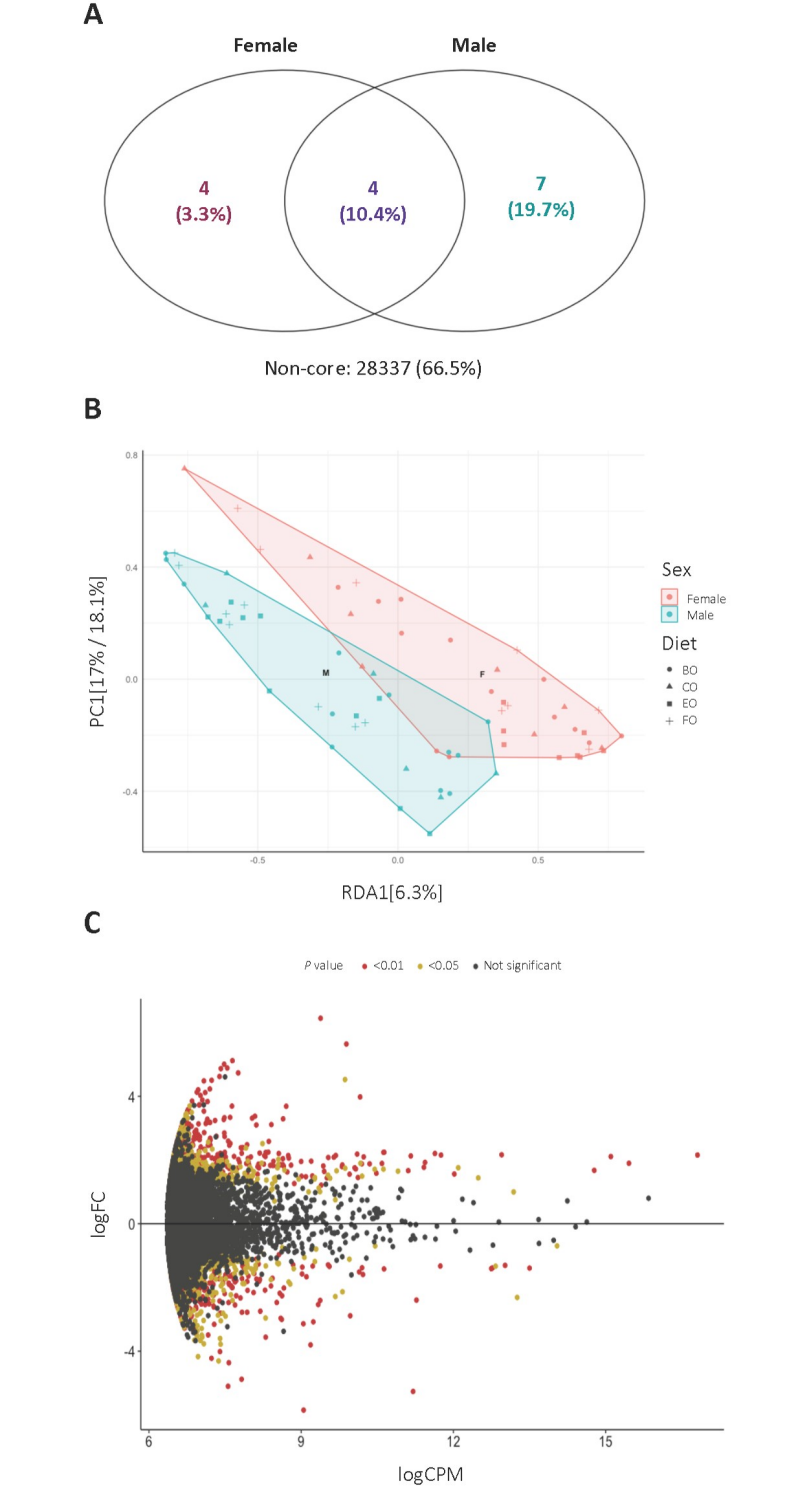
**(A) Core microbiome analysis at the operational taxonomic unit (OTU) level of the colonic bacteria in male and female CD-1 mice collapsed by diet group and age. (B) Redundancy analysis at the OTU level constrained by sex of colonic bacteria in CD-1 mice collapsed by age**. CO: CD-1 mice fed “Western-style” control fat (CO). FO: CD-1 mice fed CO supplemented with 30% fish oil. BO: CD-1 mice fed CO supplemented with 30% dairy fat. EO: CD-1 mice fed CO supplemented with 30% echium oil. **(C) M-A plot of colonic bacteria at the OTU level that were differentially abundant in males (positive y-axis) and females (negative y-axis) collapsed by diet group and age**.

To elucidate the observed sex differences in colonic bacterial composition of aged mice, analysis of colonic bacteria at the OTU level was performed. Core microbiome analysis of the average abundance of OTUs per sample showed that four colonic bacterial OTUs were specific to females, and seven colonic bacterial OTUs were specific to females; however, 66.5% of colonic bacterial abundance within individuals was not sex-specific (Fig 4A). A redundancy analysis constrained by sex further demonstrated a high variability in colonic bacterial relative abundance within and between samples with minimal overlap; sex contributed 6.3% of the variance (Fig 4B). However, an M-A plot illustrating the log-fold change of differentially abundant OTUs revealed that 73 OTUs were differentially abundant between males and females (false discovery rate < 0.05; Fig 4C). The differentially abundant OTUs, mapped to the species level, are included in S4 Table. A heat map of percent read abundance of the top 20 bacterial OTUs in CD-1 mice, segregated by diet, sex, and age is shown in S4 Fig. Overall, our data indicate that sex-specific differences of colonic bacterial abundance in aged mice fed diets with distinct FA compositions may be influenced by a limited number of differentially abundant bacterial taxa.

### Colonic bacterial diversity

**Fig 5:**
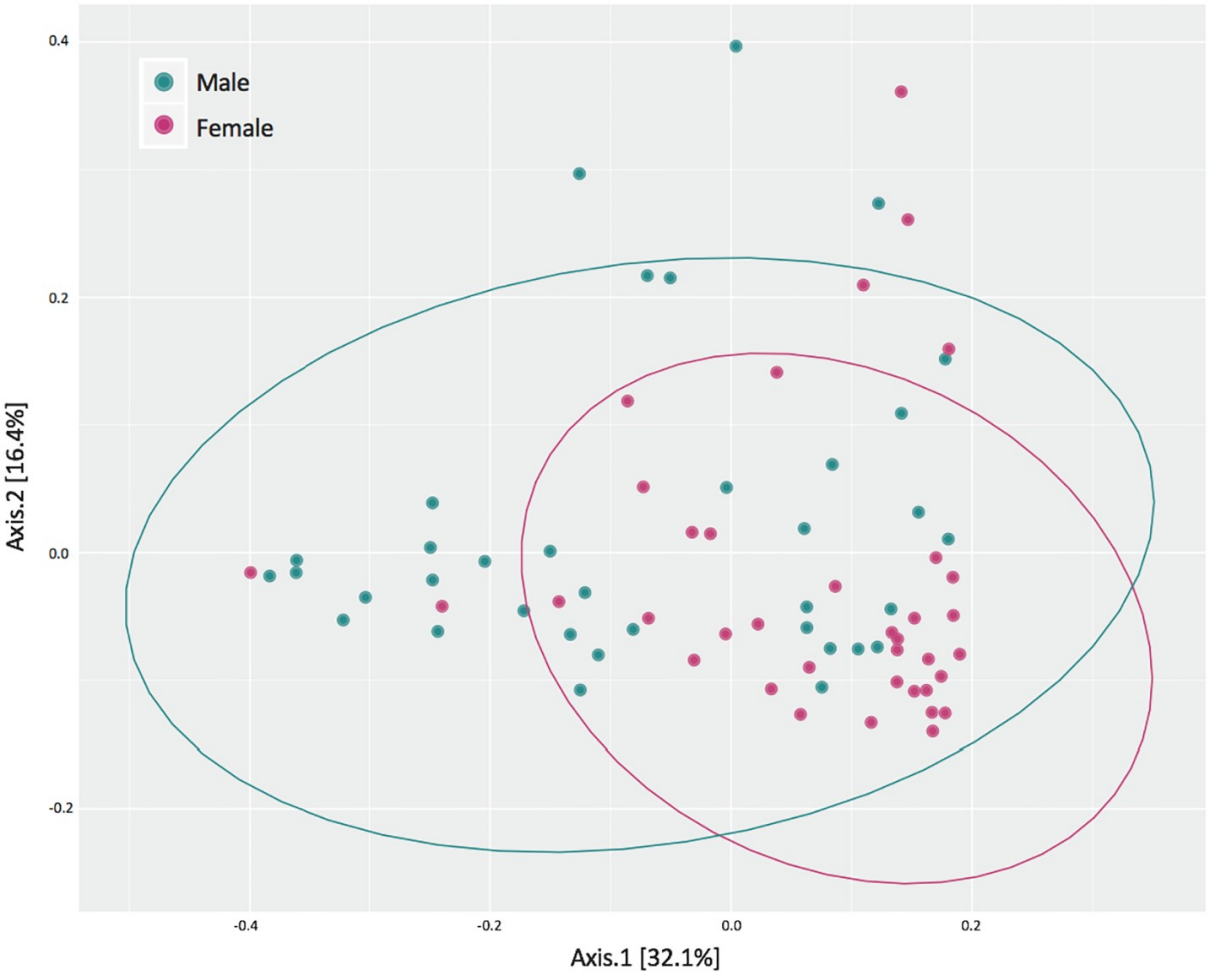
Principal coordinate analysis of beta diversity (Bray-Curtis) of male and female CD-1 mice collapsed by diet group and age.

As a covariate, body weight had no effect on colonic bacterial diversity. We detected an interactive effect between sex and age on the number of observed genera in CD-1 mice (*P* < 0.001); in females, the number of observed genera changed from 66 to 74 (10.5 and 13.5 months of age, respectively; *P* < 0.01). In addition, an interaction between diet, sex, and age (*P* < 0.05) revealed that the alpha diversity (Shannon’s Diversity Index) changed from 2.7 to 3.0 in FO-fed females, but changed from 2.6 to 2.3 in FO-fed males (10.5 versus 13.5 months of age, respectively). The colonic bacterial alpha diversity measurements for diet groups, collapsed by sex and age, are shown in S5 Table. Beta diversity (*i*.*e*., Bray-Curtis) was greater in males compared to females (*P* < 0.05; Fig 5). Taken together, these results provide evidence that aged males and females exhibit differences in colonic bacterial diversity.

## Discussion

The purpose of this study was to examine if there is a sex-dependent effect of a long-term dietary intervention with distinct FA constituents on the colonic bacteria in an aged, outbred mouse population. In mice, it has been reported that a high-fat “Western-style” diet increases the relative abundance of *Firmicutes* and decreases the relative abundance of *Bacteroidetes* [18–20], a gut bacterial composition that has been associated with several metabolic abnormalities [21–24,38,41]. We found that mice consuming a diet supplemented with echium oil had a greater abundance of *Bacteroidetes* than mice fed a “Western-style” control fat. While there are only limited studies on the role of dietary fat quality in gut bacterial composition, de Wit et al. [22] demonstrated that mice fed a 45% fat (of total energy) diet of safflower oil rich in PUFA had a greater abundance of *Bacteroidetes* compared to mice fed palm oil. Byerley et al. [42] found that male rats fed a diet containing walnuts (18% of total energy), a dietary source rich in the PUFA *α*-linolenic acid (~14% of total FA) [43], exhibited a reduced the abundance of *Bacteroidetes* compared to rats fed a control diet without walnuts; yet, whether these changes were due to *α*-linolenic or another component of walnuts is unclear. Our study is the first to characterize the effects of echium oil, a unique plant (seed) oil, on colonic microbiota structure. Echium oil contains a significant proportion of PUFA, including *α*-linolenic acid, stearidonic acid, and *γ*-linolenic acid (S6 Table). Although no studies have yet been conducted, it is conceivable that intake of these unique FA constituents may directly or indirectly modulate gut bacterial composition (*e*.*g*., changes in bile acid metabolism).

In humans, we have previously demonstrated that a diet supplemented with dairy fat reduced the ratio of *Firmicutes* to *Bacteroidetes* compared to a control diet lacking dairy fat [44]. In the current study, we did not detect differences in the *Firmicutes* to *Bacteroidetes* ratio among the different diet groups. However, we observed an interaction between the effects of diet, sex, and age whereby in BO- and EO-fed mice, the abundance of *Bacteroidetes*, as well as the genus *Bacteroides*, changed inversely in males and females as aging progressed. While these observations are not consistent with the current literature, this pattern can likely be attributed to different study designs. In this study, the dietary fat sources of interest (fish oil, dairy fat, or echium oil, respectively) were supplemented at 30% of total fat content, while other studies used 80–100% of an experimental fat source [21,22,28]. Although the experimental fat blends were intentionally formulated to reflect realistic human dietary patterns, this relatively conservative supplementation may have attenuated the potential impact of these dietary fat sources to induce colonic bacterial shifts. Additionally, other studies utilized inbred strains of mice, while this study intentionally employed an outbred stock to reflect human genetic variation. Hence, genetic diversity may be the reason why dietary fat quality did not have a pronounced effect on the colonic bacterial composition in this study. Another potential factor contributing to the inconsistency between results from other studies may be variability in resident colonic bacteria present among different animal husbandry facilities [45].

We also hypothesized that a high-fat diet supplemented with unique FA would enrich the diversity of colonic bacteria compared to a “Western-style” control fat. Greater bacterial diversity is considered to be beneficial to host health by rendering the host more resilient to gastrointestinal insults and disease [46]. Thus far, results from mouse models suggest that diets higher in total SFA may reduce bacterial diversity [22], while studies in both mice and humans have indicated that diets rich in PUFA enhance bacterial diversity [22,47]. Specifically, supplementation of the diet with fish oil, rich in PUFA such as eicosapentaenoic acid and docosahexaenoic acid, has been shown to enhance alpha diversity in mice [48]. In our study, we also found that alpha diversity (Shannon’s Diversity Index) of colonic bacteria increased with age in females fed a diet supplemented with fish oil (consisting of 40% PUFA; Table 4). Yet, no differences in alpha diversity of colonic bacteria were observed in mice fed a diet supplemented with echium oil (consisting of 72% PUFA; Table 4). Although there is limited research to distinguish the effects of dietary fats with different PUFA compositions on gut bacterial composition, these results suggest the possibility that consumption of very long-chain n-3 PUFA (*e*.*g*., docosahexaenoic acid) may have a more potent influence on colonic bacterial diversity than consumption of shorter chain n-3 PUFA (*e*.*g*., *α*-linolenic acid), either directly or indirectly.

**Table 4:** Composition of experimental diets.

Diet component	Diet			
	CO	FO	BO	EO
	*g/kg diet*			
**Basal diet mix**^1^	790	790	790	790
**Fiber**	50	50	50	50
**U.S. fat blend**^2^	210	147	147	147
**Fish oil supplement**^3^	-	63	-	-
**Dairy fat supplement**	-	-	63	-
**Echium oil supplement**	-	-	-	63
	*% of energy*			
**Protein**	17	17	17	17
**Carbohydrate**	43	43	43	43
**Fat**	40	40	40	40
**Fatty acid composition**	*g/100g total fatty acids*			
Σ SFA^4^	37.32	33.51	67.05	10.29
Σ MUFA^5^	39.69	21.98	26.66	15.77
Σ PUFA^6^	22.82	39.93	4.52	71.88
**Key fatty acids**				
Σ SCFA^7^	0.57	0.00	6.16	0.00
Σ OBCFA^8^	0.29	1.92	4.17	0.10
18:1 *t*11	0.03	0.00	1.84	0.00
Σ CLA^9^	0.07	0.03	0.87	0.00
Σ n-3	2.36	31.17	0.99	48.28
18:3 *c*9,*c*12,*c*15	2.27	1.77	0.81	34.48
18:4 *c*6,*c*9,*c*12,*c*15	0.00	2.16	0.00	13.39
20:4 *c*8,*c*11,*c*14,*c*17	0.00	1.08	0.04	0.00
20:5 *c*5,*c*8,*c*11,*c*14,*c*17	0.00	14.80	0.06	0.00
22:5 *c*7,*c*10,*c*13,*c*16,*c*19	0.03	2.63	0.10	0.00
22:6 *c*4,*c*7,*c*10,*c*13,*c*16,*c*19	0.00	9.56	0.00	0.00
Σ n-6	20.31	3.96	1.82	23.58
18:2 *c*9,*c*12	19.66	1.18	1.61	13.86
18:3 *c*6,*c*9,*c*12	0.01	0.29	0.02	9.65
20:4 *c*5,*c*8,*c*11,*c*14	0.14	1.32	0.10	0.00
n-6/n-3 ratio	8.6	0.1	1.8	0.5

^1^Supplied and formulated by Research Diets Inc.

^2^U.S. fat blend consisted of lard, walnut oil, high-oleic sunflower oil, coconut oil, and palm oil in a ratio of 18.8:3.6:2.8:1.8:1.0.

^3^Fish oil supplement is derived from menhaden.

^4^SFA: saturated fatty acids.

^5^MUFA: monounsaturated fatty acids.

^6^PUFA: polyunsaturated fatty acids.

^7^SCFA: short-chain fatty acids (4:0–8:0).

^8^OBCFA: odd- and branched-chain fatty acids (5:0; 7:0; 9:0; 11:0; 13:0; 13:0 *iso*; 13:0 *aiso*; 14:0 *iso*; 15:0; 15:0 *iso*; 15:0 *aiso*; 15:0; 16:0 *iso*; 17:0; 17:0 *iso*; 17:0 *aiso*; 18:0 *iso*; 17:1 *c*8; 17:1 *c*9; 17:1 *t*10; 19:0; 21:0; 23:0).

^9^CLA: conjugated linoleic acids (18:2 *t*7/*t*9/*t*10,*t*12; 18:2 *c*8,*c*10; 18:2 *t*8,*c*10/*t*7,*c*9; 18:2 *c*9,*c*11; 18:2 *c*9,*t*11; 18:2 *t*9,*c*11; 18:2 *c*10,*c*12; 18:2 *t*10,*c*12; 18:2 *c*11,*c*13; 18:2 *t*11,*t*13).

Studies have shown that, in general, a greater dietary diversity enriches gut bacterial diversity, likely due to enhanced substrate utilization and opportunity of cross-feeding among bacteria [49]. Moreover, it has been postulated that an increase in dietary FA diversity would also induce an enrichment of bacterial diversity [49]. This may also explain in part why the FO diet, comprising a greater FA diversity, increased colonic bacterial diversity compared to the CO diet. For this reason, we hypothesized that the BO diet, despite its high SFA content, would also lead to a greater colonic bacterial diversity due to the greater FA diversity (*i*.*e*., short-, odd-, and branched-chain FA; vaccenic acid; conjugated linoleic acids; Table 4). Yet, BO-fed mice did not exhibit an enhanced bacterial diversity compared to CO-fed mice. Despite the unique FA diversity of the BO diet, the concentration of these FA in the diet may have been too low to counterbalance the prevailing influence of the SFA in the BO diet to maintain bacterial diversity. The BO diet contained almost twice the SFA content compared to the CO diet (67% SFA compared to 37% SFA, respectively; Table 4). Thus, consistent with previous work [22], it is plausible that no detectable changes in bacterial diversity were could be observed due to the high content of SFA in the BO diet.

Similar to other studies, our study specifically investigated the relationship between body weight and colonic bacterial community structure. Because mice are coprophagous, and when caged in pairs, we expected for cage mates to be similar in gut bacterial community structure. As several studies suggest that body weight is intimately associated with gut bacterial composition [20,23–27,40], by extension, this indicates that mice housed together under the same dietary regime should also be similar in weight. However, our data revealed that body weight had limited effect colonic bacteria at the genus level. Moreover, neither the abundance of at the phylum level nor diversity of colonic bacteria were associated with body weight, despite considerable variation in the weight of cage mates. Thus, these observations point to the alternative hypothesis that obesity development may be independent of gut bacterial composition [19,50,51]. As we studied outbred CD-1 mice, a genetically heterogeneous stock, it is possible that other inherent factors, such as genetic diversity, contributed to the differences in body weight. In addition, a limitation of our study is that we were only able to measure feed intake on a cage basis; hence, feed intake may have varied between cage mates.

Aging has been identified as a contributing factor in shaping the composition of gut bacteria, but only few longitudinal studies have been performed. Observational studies in humans revealed an increase in relative abundance of *Bacteroidetes* [12,52,53] and loss in bacterial diversity [54] following the transition to the geriatric age. However, confounding variables among individuals such as diet, geographic region, and past experience (*e*.*g*., medical history) result in lack of a consistent pattern. Mouse studies have also reported conflicting results of age-related changes in the gut microbiota. Fransen et al. [15] reported a decreased ratio of *Firmicutes* to *Bacteroidetes* in aged mice (18 months old) compared to young mice (3 months old). Another study found no differences in the relative abundance of *Firmicutes* or *Bacteroidetes* but an increased alpha diversity and a variable beta diversity in aged mice (19 months old) compared to young mice (3 months old) [55]. While there were no changes in beta diversity in mice at a mid-life compared to an aged life stage in our study, alpha diversity (*i*.*e*., number of observed genera) increased in female mice with age. This study did not examine the colonic bacteria of frank geriatric mice (considered to be 18 months of age) due to advanced morbidity in our CD-1 mouse population investigated; however, our data further demonstrate that sex may, at least in part, influence the gut microbiota community structure in aged mice. More research is warranted to determine whether sex may play a role in modulating the effects of dietary fat quality on the colonic bacterial community structure in the frank geriatric population.

Recent evidence implicates sex playing a role in gut bacterial composition [32–34], although the underlying mechanisms are not fully understood. Yurkovetskiy et al. [56] examined i) male, female, and castrated male NOD mice at pre- and post-pubescent ages, and ii) specific pathogen-free versus germ-free NOD mice, and concluded that androgens and the gut microbiota contribute equally to host health in the context of autoimmune diabetes. Org et al. [35] utilized gonadectomized mice and determined that the difference in circulating androgens between males and females contributed to microbiota changes. In addition, sex-dependent associations of gut bacteria with disease conditions have recently been reported in humans [57,58]. Although our study did not incorporate gonadectomized mice, we also verified that sex is a key determinant of the colonic bacterial community structure in aged mice. In our study, sex was observed to affect bacterial diversity as aging progressed. In addition, we found that 73 colonic bacterial OTUs were differentially abundant in males versus females. This suggests that the greater abundance of *Bacteroidetes* and a lower ratio of *Firmicutes* to *Bacteroidetes* observed in females compared to males, respectively, may have been driven by a select number of colonic bacteria at the OTU level. In our study, there was a high level of variability between and within colonic bacterial samples as highlighted by redundancy analysis (Fig 4B). This variability, likely stemming from the genetic diversity inherent in the CD-1 mouse stock, is the probable underlying factor that prevented more pronounced differences in colonic bacterial composition between males and females. Sampling a larger population may have elucidated more profound sex-specific differences in these heterogenous, aged mice. Overall, these results further emphasize the effect that sex of the host and, by inference, the sex hormone milieu, can exert on the gut bacterial community structure.

The manner in which dietary patterns influence the gut bacteria is increasingly gaining interest, as the gut bacterial composition is now known to significantly contribute to an individual’s health status. Our work with the outbred CD-1 mouse model supports the hypothesis that sex is an important variable in colonic bacterial composition in an aged, genetically diverse population. Moreover, results from this study demonstrate a strong interactive effect between the diet, sex, and age of mice on the colonic bacterial community structure. Thus, our findings establish that there is an intricate relationship between an individual’s sex, age, and genetic background that collectively factors into the susceptibility of the gut bacteria to undergo population changes in response to diet. While our study solely assessed modifications to the colonic bacterial composition, such changes are indicative of its derived metabolites that affect the systemic metabolism of the host. Accordingly, this study has important implications for the development of nutritional strategies to maintain health and, by extension, mitigate risk of disease in humans. Moving forward, further research is needed to ascertain whether dietary fat quality shapes the gut bacterial composition in a direct or indirect manner, and whether and to what degree host factors facilitate the interaction pathways.

## Methods

### Animals and experimental design

All procedures were approved by and performed in accordance with guidelines and regulations of The University of Vermont Institutional Animal Care and Use Committee (#16–007). Eighty-one male and female outbred mice (CD-1^®^ IGS #022) were purchased from Charles River (Raleigh, NC, USA), an ideal aging rodent model with a genetic variability comparable to the human population compared to other inbred strains [59–61]. At the age of three weeks, mice arrived in two shipping boxes, segregated by sex, and were immediately randomized into cages. Mice were housed in ventilated racks (Thorens Caging Systems, Hazelton, PA, USA) in same-sex pairs, with the exception of one group of three, on a 12-hour light/dark cycle at 23.6°C with 64% humidity. Mice were adjusted to their new environment for one week; during this time mice were fed a laboratory chow diet consisting of 26% protein, 60% carbohydrate, and 14% fat (Prolab^®^ Rat/Mouse/Hamster 3000, LabDiet, St. Louis, MO, USA). After acclimatization, mice (n = 10-11/group) were fed one of the following high-fat (40% kcal) diets for 13 months: i) CO: 100% control fat (U.S. fat blend), ii) FO: 70% CO + 30% fish (menhaden) oil, iii) BO: 70% CO + 30% dairy (butter) fat, or iv) EO: 70% CO + 30% echium oil; Table 4).

Mice were provided with feed and water *ad libitum* for the entirety of the study. Body weight was recorded weekly until three months of age, and thereafter every four weeks for the remainder of the study. Feed intake was determined on a cage basis at weekly intervals.

### Experimental diets

Diets were identical with the exception of the FA composition. All fat blends, except for the addition of fish oil, were prepared in-house. The U.S. fat blend was designed of lard, walnut oil, high-oleic sunflower oil, coconut oil, and palm oil in a ratio of 18.8:3.6:2.8:1.8:1.0 to reflect the FA profile of an average U.S. American diet [39] (Table 4). Pure butter fat was separated from water through gentle heating of butter into a liquid phase and subsequent centrifugation at 3,434 *g* at 4°C for 60 min (Sorvall Legend RT Centrifuge, ThermoFisher Scientific, Waltham, MA, USA) to yield a solid supernatant of pure butter fat. Echium oil was procured from Technology Crops International (Winston-Salem, NC, USA). Dairy fat and echium oil replaced 30% (by weight) of U.S. fat blend to produce BO and EO fat blends, respectively. Fat blends were sent to Research Diets, Inc. (Brunswick, NJ, USA) to be incorporated into the diets (pelleted form). In the case of the fat blend supplemented with fish oil, menhaden oil (30% final weight) was blended into the U.S. fat blend on-site at Research Diets, Inc. All experimental diets were isoenergetic and consisted of 17% protein, 43% carbohydrates, and 40% fat (Table 4). The complete FA composition of the experimental fat supplements is shown in S6 Table.

### Fatty acid analysis of experimental fat blends

Experimental fat blends were prepared as FA methyl esters and analyzed via gas-liquid chromatography analysis following the method described by Bainbridge et al. [62].

### Fecal collection and DNA extraction

Fecal collection was carried out at 10.5 and 13.5 months of age to represent the colonic bacterial community structure at mid-life (adult) and aged (reproductively senescent) life stages, respectively. Due to logistical reasons *(e*.*g*., number of metabolism cages), two cages (cage mates in sets of two or three; n = 4-5/sex/diet group) were selected for analysis of the colonic bacteria. Morbidity of one CO-fed male and poor DNA quality after isolation from a fecal sample of another CO-fed male caused two rather than four samples to be analyzed at 10.5 months of age. At 13.5 months of age, fecal samples were collected from an additional nine mice fed dairy fat (n = 8-9/sex/BO diet). Our secondary objective was to examine the effect of body weight on the colonic bacterial composition; obesity has previously been associated with changes in gut microbiota community structure [20,23–26,40]. Accordingly, mice that were selected for analysis of colonic bacterial composition included cage mates disparate in weight when possible (*e*.*g*., 26 g versus 56 g).

Fecal samples were collected in 70% ethanol over a 24-hour period via individual metabolism cages (Techniplast, West Chester, PA, USA) and stored at 4°C for subsequent DNA extraction. Total microbial DNA was extracted via QIAmp DNA Stool Mini Kit (Qiagen, Germantown, MD, USA) following the method of Yu and Morrison [63]. Modifications described in Cersosimo et al. [64] were followed with an additional modification of using 50% more InhibitEx per sample. The DNA extract was verified for adequate concentration and purity via Nanodrop 2000c Spectrophotometer (ThermoFisher Scientific, Waltham, MA, USA) and stored at -20°C until PCR amplification.

### PCR amplification

The V1-V3 region of the 16S rRNA gene was amplified using the bacteria-specific primer pair 27F and 519R (Integrated DNA Technologies, Skokie, IL, USA) on the GeneAmp PCR System 9700 (Applied Biosystems, Foster City, CA, USA). Each PCR reaction contained 50 μL total volume with 2 μL of extracted DNA (diluted to 10 ng/μL). The PCR program consisted of the following: hot start at 98°C held for 3 min; 30 cycles of 98°C for 30 sec, 56°C for 30 sec, 72°C for 45 sec; an extension at 72°C for 3 min; then held at 8°C. A positive control of *Bacteroides vulgatus* (American Type Culture Collection, Manassas, VA, USA) and a negative control of RT-PCR Grade Water (ThermoFisher Scientific, Waltham, MA, USA) were used with all reactions. The PCR products were verified for appropriate amplicon length via gel electrophoresis and for adequate concentration via Qubit^®^ 3.0 Fluorometer (ThermoFisher Scientific, Waltham, MA, USA). Amplicons were stored at -20°C before shipment to Molecular Research DNA (Shallowater, TX, USA) for sequencing and bioinformatics analysis.

### Real-time PCR amplification

Real-time PCR was performed in triplicate via BioRad C1000 Touch Thermocycler (BioRad, Hercules, CA, USA) to determine bacterial density. Each PCR reaction contained: 12.5 μL of iQ SYBR Green Super Mix (BioRad, Hercules, CA, USA), 6.5 μL of RT-PCR Grade Water (ThermoFisher Scientific, Waltham, MA, USA), 2.5 μL of primer pair 1114F and 1275R (Integrated DNA Technologies, Skokie, IL, USA) for the 16S rRNA gene [65], and 1 μL of sample DNA. The PCR program was as follows: hot start of 98°C for 3 min; 40 cycles of 95°C for 10 sec and 60°C for 30 sec; a final melting curve analysis of the fluorescence performed between 55°C and 95°C, with increments of 0.5°C every 5 sec. Positive controls and standards of *Bacteroides vulgatus* (American Type Culture Collection, Manassas, VA, USA) in serial dilution (10 ng μL^−1^) and negative controls of RT-PCR Grade Water (ThermoFisher Scientific, Waltham, MA, USA) were used with all reactions. Only reaction plates with a serial dilution of standards that yielded an R^2^ value greater than or equal to 0.997 were included in analysis.

Determination of bacterial density did not account for variation in potential number of 16S rRNA-encoding genes per bacterium. Mean individual bacterial densities were calculated as bacterial copies per μg wet fecal pellet, under the assumption of one 16S rRNA-encoding gene per bacterium, using a previously validated method [66].

### Sequencing and bioinformatics analysis

At Molecular Research DNA (Shallowater, TX, USA), amplicons were prepared for sequencing with the addition of barcodes to forward primers of amplicons via PCR. PCR products were checked via gel electrophoresis. Samples were pooled and purified using Ampure XP beads (Agencourt Bioscience Corporation, MA, USA). Library preparation then ligated adapter sequences to the samples. Amplicons were sequenced via Illumina Miseq (v.3) following manufacturer's guidelines and sequences were processed using the MRDNA analysis pipeline. Briefly, paired-end sequences were combined and depleted of barcodes using usearch with an average length of 550 base pairs and non-overlapping reads were removed; sequences were trimmed with a quality score cut-off of 25; any sequences with <200 base pairs or with ambiguous base calls were excluded. Sequences were denoised, OTUs were generated based on 97% similarity using usearch, and chimeras were detected and removed using uchime. The mean sequencing depth was 27,536 reads, with a minimum of 9,953 and a maximum of 76,590 reads. OTUs were taxonomically classified using BLASTn top hit and compiled into text files by raw counts.

In-house, colonic bacterial abundance and diversity analyses were conducted in R version 3.5.1 using the packages *phyloseq* [67] and *ampvis2* [68] and differential OTU abundance via the package *edgeR* [69]. Normalization and transformations were conducted by normalizing to the total sequences per sample to calculate read abundance (library size) and transformed via TMM (differential OTU analysis) or Hellinger transformations for ordination. Principal coordinate analysis was performed using the package *phyloseq*. Sequencing files are available via the Sequence Read Archive (SRA) under the accession number PRJNA484010.

### Statistical analysis

Body weight, feed intake, feed efficiency, colonic bacterial density and alpha diversity, and the ratio of *Firmicutes* to *Bacteroidetes* were analyzed with PROC MIXED in SAS 9.4 (SAS Institute, Cary, NC). Colonic bacterial counts (*i*.*e*., abundance) at the phylum and genus level were analyzed with PROC GLIMMIX in SAS 9.4 (SAS Institute, Cary, NC). Poisson or negative binomial distribution was assumed to provide the best fit for each dependent variable, determined by whether or not there was over-dispersion. Both models included the fixed effects of diet, sex, and age and the random effect of cage with body weight as a covariate. Post hoc contrast statements were chosen to determine unadjusted pairwise differences due to the quantity of interactions present in the statistical model. Feed efficiency, colonic bacterial density, the ratio of *Firmicutes* to *Bacteroidetes*, and the colonic bacterial relative abundance, calculated by XXX, were graphed in GraphPad Prism version 7.00 (GraphPad Software, La Jolla, CA, USA). Differences in beta diversity (Bray-Curtis) were determined in the R package *vegan* [70] and included the factors of diet, sex, and age.

## Supporting information

S1 Table Body weight, feed intake, and feed efficiency of CD-1 mice collapsed by sex and age. Values are expressed as mean ± standard error of the mean. *P < 0.05; **P < 0.01; ***P < 0.001.
(PDF)

S2 Table Colonic bacterial density and abundance by counts at the phylum level of CD-1 mice collapsed by sex and age. Values are expressed as mean ± standard error of the mean. *P < 0.05; ***P < 0.001.
(PDF)

S3 Table Colonic bacterial abundance at the genus level of CD-1 mice collapsed by sex and age. Values are expressed as mean ± standard error of the mean. *P < 0.05; **P < 0.01; ***P < 0.001.
(PDF)

S4 Table M-A plot results of differentially abundant (*P* < 0.05) colonic bacterial OTUs in males (positive logFC) versus in females (negative logFC) collapsed by diet group and age. (XLS)

S5 Table Colonic bacterial alpha diversity measurements of male and female CD-1 mice collapsed by sex and age. Values are expressed as mean ± standard error of the mean. *P < 0.05; ***P < 0.001.
(PDF)

S6 Table Fatty acid composition of fat supplements. (PDF)

S1 Fig (A) Relative abundance of bacteria at the phylum level of male and female CD-1 mice fed “Western-style” control fat (CO) at 10.5 and 13.5 months of age. (B) Relative abundance of bacteria at the phylum level of male and female CD-1 mice fed CO supplemented with 30% fish oil (FO) at 10.5 and 13.5 months of age. (C) Relative abundance of bacteria at the phylum level of male and female CD-1 mice fed CO supplemented with 30% dairy fat (BO) at 10.5 and 13.5 months of age. (D) Relative abundance of bacteria at the phylum level of male and female CD-1 mice fed CO supplemented with 30% echium oil (EO) at 10.5 and 13.5 months of age.
(PDF)

S2 Fig Relative abundance of bacteria at the genus level of male and female CD-1 mice collapsed by diet group and age. (PDF)

S3 Fig (A) Relative abundance of bacteria at the genus level of male and female CD-1 mice fed “Western-style” control fat (CO) at 10.5 and 13.5 months of age. (B) Relative abundance of bacteria at the genus level of male and female CD-1 mice fed CO supplemented with 30% fish oil (FO) at 10.5 and 13.5 months of age. (C) Relative abundance of bacteria at the genus level of male and female CD-1 mice fed CO supplemented with 30% dairy fat (BO) at 10.5 and 13.5 months of age. (D) Relative abundance of bacteria at the genus level of male and female CD-1 mice fed CO supplemented with 30% echium oil (EO) at 10.5 and 13.5 months of age.
(PDF)

S4 Fig Heat map of read abundance of the 20 most abundant colonic bacterial OTUs in CD-1 mice separated by diet, sex, and age. (PDF)
