# Supplementary material for: Colonic bacterial composition is sex-specific in aged CD-1 mice fed diets varying in fat quality
Source: PLoS One. 2019 Dec 18;14(12):e0226635. doi: 10.1371/journal.pone.0226635 (PMC6919604; doi:10.1371/journal.pone.0226635)
Supplement: S1 Table — Values are expressed as mean ± standard error of the mean. *P < 0.05; **P < 0.01; ***P < 0.001. (PDF) [file pone.0226635.s001.pdf]

S1 Table. Body weight, feed intake, and feed efficiency of CD-1 mice collapsed by sex and age. Values are expressed as mean ± standard error of the mean. $^{*}P < 0.05$; $^{**}P < 0.01$; $^{***}P < 0.001$.

									P value						
**Parameter**	**CO[1]**	**SEM**	**FO[2]**	**SEM**	**BO[3]**	**SEM**	**EO[4]**	**SEM**	**D[5]**	**S[6]**	**A[7]**	**D*S**	**D*A**	**S*A**	**D*S*A**
**Body weight (g)**	44.1	2.7	56.7	4.9	50.9	3.5	51.2	3.6	-	***	*	-	-	-	-
**Feed intake (g/day)**	3.1	0.2	3.6	0.1	3.3	0.1	3.4	0.0	-	-	**	-	**	-	-
**Feed efficiency[8]**	0.005	0.000	0.006	0.001	0.005	0.001	0.005	0.001	-	***	***	-	-	***	-

[1]CO: CD-1 mice fed a "Western-style" control fat. [2]FO: CD-1 mice fed CO supplemented with 30% fish oil. [3]BO: CD-1 mice fed CO supplemented with 30% dairy fat. [4]EO: CD-1 mice fed CO supplemented with 30% echium oil. [5]D: Diet. [6]S: Sex. [7]A: Age. [8]Final body weight (g) – initial body weight (g) / total feed (kcal) consumed.
