# Supplementary material for: Colonic bacterial composition is sex-specific in aged CD-1 mice fed diets varying in fat quality
Source: PLoS One. 2019 Dec 18;14(12):e0226635. doi: 10.1371/journal.pone.0226635 (PMC6919604; doi:10.1371/journal.pone.0226635)
Supplement: S2 Table — Values are expressed as mean ± standard error of the mean. *P < 0.05; ***P < 0.001. (PDF) [file pone.0226635.s002.pdf]

S2 Table. Colonic bacterial density and abundance by counts at the phylum level[1] of CD-1 mice collapsed by sex and age. Values are expressed as mean ± standard error of the mean. $^{*}P < 0.05$; $^{***}P < 0.001$.

									P value						
	**CO[2]**	**SEM**	**FO[3]**	**SEM**	**BO[4]**	**SEM**	**EO[5]**	**SEM**	**D[6]**	**S[7]**	**A[8]**	**D*S**	**D*A**	**S*A**	**D*S*A**
**Density[9]**	14.0	0.1	14.1	0.1	14.3	0.1	14.1	0.1	-	-	***	-	-	-	-
**Phylum**															
Bacteroidetes	4613	594	5234	657	8316	1444	9248	1767	*	*	-	-	-	-	***
Firmicutes	17979	3098	22701	4832	16337	2274	20108	3744	-	-	-	-	-	*	-
Proteobacteria	255	65	499	130	1096	670	192	58	-	-	-	-	-	-	-
Verrucomicrobia	173	68	128	66	1193	423	536	388	-	-	-	-	-	-	-
***Firmicutes:Bacteroidetes* ratio**	5.7	1.7	6.3	1.9	4.3	1.0	5.5	2.0	-	*	-	-	-	-	-

[1]Mean relative abundance > 1%. [2]CO: CD-1 mice fed a "Western-style" control fat. [3]FO: CD-1 mice fed CO supplemented with 30% fish oil. [4]BO: CD-1 mice fed CO supplemented with 30% dairy fat. [5]EO: CD-1 mice fed CO supplemented with 30% echium oil. [6]D: Diet. [7]S: Sex. [8]A: Age. [9]Bacterial log copies per µg fecal pellet.
