# Supplementary material for: Colonic bacterial composition is sex-specific in aged CD-1 mice fed diets varying in fat quality
Source: PLoS One. 2019 Dec 18;14(12):e0226635. doi: 10.1371/journal.pone.0226635 (PMC6919604; doi:10.1371/journal.pone.0226635)
Supplement: S3 Table — Values are expressed as mean ± standard error of the mean. *P < 0.05; **P < 0.01; ***P < 0.001. (PDF) [file pone.0226635.s003.pdf]

S3 Table. Colonic bacterial abundance by counts at the genus level[1] of CD-1 mice collapsed by sex and age. Values are expressed as mean ± standard error of the mean. $^{*}P < 0.05$; $^{**}P < 0.01$; $^{***}P < 0.001$.

									P value						
**Genus**	**CO[2]**	**SEM**	**FO[3]**	**SEM**	**BO[4]**	**SEM**	**EO[5]**	**SEM**	**D[6]**	**S[7]**	**A[8]**	**D*S**	**D*A**	**S*A**	**D*S*A**
Akkermansia	173	68	128	66	1193	423	536	388	-	-	-	-	-	-	-
Alistipes	698	215	933	198	1207	134	1855	385	-	-	-	-	-	***	*
Allobaculum	2491	1761	3351	1492	1457	516	1414	789	-	-	-	-	-	-	-
Anaerostipes	397	179	313	104	308	50	392	85	-	-	-	-	-	-	-
Bacteroides	2558	381	3111	421	5307	1256	5210	1363	-	-	-	-	-	-	*
Barnesiella	534	184	672	197	486	145	885	345	-	-	***	-	-	**	-
Bilophila	231	69	493	129	286	82	182	58	-	-	*	-	-	*	-
Blautia	590	178	353	58	458	48	624	104	-	-	-	-	-	-	-
Clostridium	6751	1324	6749	1302	5805	743	8023	1509	-	-	-	-	-	-	-
Eubacterium	489	141	268	61	177	35	302	68	-	-	-	-	-	-	-
Lachnoclostridium	377	96	269	46	325	35	412	51	-	-	-	-	-	-	-
Lactobacillus	3687	1130	8663	3110	3916	1356	4300	1533	-	-	-	-	-	-	-
Oscillospira	760	139	710	145	789	106	920	130	-	-	-	-	-	-	-
Parabacteroides	784	201	474	72	1281	283	1099	327	-	-	-	-	-	-	-
Roseburia	466	118	366	68	394	47	541	102	-	*	-	-	-	-	-
Ruminococcus	571	189	167	36	241	70	1003	639	-	-	-	-	-	*	-
Turicibacter	302	122	632	202	1195	349	826	350	-	-	**	-	-	*	-

[1]Mean relative abundance > 1%. [2]CO: CD-1 mice fed a "Western-style" control fat. [3]FO: CD-1 mice fed CO supplemented with 30% fish oil. [4]BO: CD-1 mice fed CO supplemented with 30% dairy fat. [5]EO: CD-1 mice fed CO supplemented with 30% echium oil. [6]D: Diet. [7]S: Sex. [8]A: Age.
