# Supplementary material for: Colonic bacterial composition is sex-specific in aged CD-1 mice fed diets varying in fat quality
Source: PLoS One. 2019 Dec 18;14(12):e0226635. doi: 10.1371/journal.pone.0226635 (PMC6919604; doi:10.1371/journal.pone.0226635)
Supplement: S5 Table — Values are expressed as mean ± standard error of the mean. *P < 0.05; ***P < 0.001. (PDF) [file pone.0226635.s005.pdf]

S5 Table. Colonic bacterial alpha diversity measurements of male and female CD-1 mice collapsed by sex and age. Values are expressed as mean ± standard error of the mean. $^{*}P < 0.05$; $^{***}P < 0.001$.

									P value						
**Alpha diversity measurement**	**CO[1]**	**SEM**	**FO[2]**	**SEM**	**BO[3]**	**SEM**	**EO[4]**	**SEM**	**D[5]**	**S[6]**	**A[7]**	**D*S**	**D*A**	**S*A**	**D*S*A**
**Number of observed genera**	66	3	68	2	70	2	78	3	-	-	-	-	-	***	-
**Shannon's Diversity Index**	2.6	0.1	2.6	0.1	2.7	0.1	2.7	0.1	-	-	-	-	-	*	*

[1]CO: CD-1 mice fed a "Western-style" control fat. [2]FO: CD-1 mice fed CO supplemented with 30% fish oil. [3]BO: CD-1 mice fed CO supplemented with 30% dairy fat. [4]EO: CD-1 mice fed CO supplemented with 30% echium oil. [5]D: Diet. [6]S: Sex. [7]A: Age.
