# Supplementary material for: Colonic bacterial composition is sex-specific in aged CD-1 mice fed diets varying in fat quality
Source: PLoS One. 2019 Dec 18;14(12):e0226635. doi: 10.1371/journal.pone.0226635 (PMC6919604; doi:10.1371/journal.pone.0226635)
Supplement: S6 Table — (PDF) [file pone.0226635.s006.pdf]

S6 Table. Fatty acid composition of fat supplements.

	Fat supplement			
Fatty acid	**CO**[1]	**FO**	**BO**	**EO**
	g/100g fatty acids			
4:0	0.00	0.00	3.10	0.00
5:0	0.00	0.00	0.03	0.00
6:0	0.05	0.00	2.01	0.00
7:0	0.00	0.00	0.02	0.00
8:0	0.59	0.00	1.20	0.00
9:0	0.00	0.00	0.04	0.00
10:0	0.49	0.00	2.81	0.00
11:0	0.00	0.00	0.32	0.00
11 cyclohexyl/11:0	0.00	0.00	0.04	0.00
12:0	3.25	0.11	3.31	0.00
13:0	0.00	0.05	0.21	0.00
13:0 *iso*	0.00	0.01	0.02	0.00
13:0 *aiso*	0.00	0.00	0.08	0.00
14:0	2.08	8.40	10.72	0.03
14:0 *iso*	0.00	0.06	0.10	0.00
14:1 *c*9	0.00	0.04	0.89	0.00
15:0	0.04	0.65	1.17	0.00
15:0 *iso*	0.00	0.29	0.21	0.00
15:0 *aiso*	0.00	0.13	0.44	0.00
16:0	20.04	14.61	29.87	6.87
16:0 *iso*	0.00	0.12	0.28	0.00
16:1 *c*7	0.18	0.21	0.14	0.00
16:1 *c*9	1.23	12.04	1.34	0.07
16:1 *t*9	0.00	0.20	0.05	0.00
16:1 *c*11	0.00	0.19	0.03	0.00
16:1 isomer	0.00	0.00	0.04	0.00
16:2 *c*9,*c*12	0.00	1.39	0.00	0.00
16:3 *c*6,*c*9,*c*12	0.00	1.56	0.00	0.00
17:0	0.24	0.46	0.60	0.10
17:0 *iso*	0.00	0.00	0.32	0.00
17:0 *aiso*	0.00	0.00	0.40	0.00
17:1 *c*8	0.00	0.07	0.00	0.00
17:1 *c*9	0.00	0.12	0.00	0.00
17:1 *t*10	0.00	0.26	0.00	0.00
18:0	10.30	2.49	10.44	3.52
18:0 *iso*	0.00	0.27	0.07	0.00
18:1 *t*4	0.00	0.00	0.04	0.00
18:1 *t*5	0.00	0.00	0.02	0.00
18:1 *t*6-8	0.00	0.00	0.29	0.00
18:1 *c*9	36.93	3.58	19.67	14.89
18:1 *t*9	0.00	0.00	0.25	0.00
18:1 *t*10	0.00	0.00	0.45	0.00
18:1 *c*11	1.64	2.44	0.66	0.47
18:1 *t*11	0.00	0.00	1.20	0.00
18:1 *c*12	0.00	0.00	0.46	0.00
18:1 *t*12	0.00	0.00	0.45	0.00
18:1 *c*13	0.05	0.05	0.11	0.00

18:1 *c*14/*t*16	0.00	0.00	0.38	0.00
18:1 t13/*t*14	0.00	0.00	0.78	0.00
18:1 *c*15	0.00	0.79	0.06	0.00
18:1 *c*16	0.00	0.00	0.08	0.00
18:2 *c*9,*t*11	0.00	0.00	0.51	0.00
18:2 *c*9,*c*12	19.51	1.28	2.48	14.15
18:2 *c*9,*t*12	0.00	0.00	0.00	0.14
18:2 *c*9,*t*13/*t*8,*c*12	0.00	0.00	0.21	0.00
18:2 *c*9,*t*14	0.00	0.00	0.09	0.00
18:2 *t*9,*c*12	0.00	0.00	0.00	0.02
18:2 *t*10,*t*14	0.00	0.00	0.06	0.00
18:2 *t*11,*t*13	0.00	0.00	0.00	0.00
18:2 *t,t* isomer	0.00	0.00	0.00	0.00
18:3 *c*6,*c*9,*c*12	0.00	0.32	0.00	10.01
18:3 *c*9,*c*12,*c*15	1.89	1.29	0.39	32.71
18:3 *c*9,*t*12,*c*15	0.00	0.00	0.00	0.06
18:3 *c*9,*t*12,*t*15/*c*9,*c*12,*t*15	0.00	0.00	0.00	0.70
18:3 *t*9,*c*12,*c*15	0.00	0.00	0.00	0.50
18:4 *c*6,*c*9,*c*12,*c*15	0.00	2.57	0.00	13.38
19:0	0.01	0.07	0.00	0.00
20:0	0.24	0.14	0.15	0.10
20:1 *c*9	0.00	0.42	0.10	0.00
20:1 *c*11	0.42	0.00	0.03	0.74
20:2 *c*11,*c*14	0.41	0.17	0.00	0.05
20:3 *c*5,*c*8,*c*11	0.06	0.29	0.13	0.00
20:3 *c*11,*c*14,*c*17	0.00	0.24	0.00	0.00
20:4 *c*5,*c*8,*c*11,*c*14	0.14	1.36	0.16	0.00
20:4 *c*8,*c*11,*c*14,*c*17	0.00	1.19	0.00	0.00
20:5 *c*5,*c*8,*c*11,*c*14,*c*17	0.00	13.29	0.03	0.00
22:0	0.05	0.15	0.06	0.05
22:1 *c*13	0.05	0.00	0.00	0.39
22:1 *t*13	0.00	0.00	0.00	0.05
22:2 *c*13,*c*16	0.00	0.03	0.00	0.00
23:0	0.00	0.05	0.00	0.00
24:0	0.03	0.07	0.00	0.00
24:1 *c*15	0.01	0.25	0.00	0.11
22:4 *c*7,*c*10,*c*13,*c*16	0.07	0.21	0.00	0.00
22:5 *c*4,*c*7,*c*10,*c*13,*c*16	0.00	0.54	0.00	0.00
22:5 *c*7,*c*10,*c*13,*c*16,*c*19	0.00	2.02	0.00	0.00
22:6 *c*4,*c*7,*c*10,*c*13,*c*16,*c*19	0.00	10.33	0.00	0.00
Unknown	0.00	3.12	0.43	0.89

[1]CO: U.S. fat blend consisted of lard, walnut oil, high-oleic sunflower oil, coconut oil, and palm oil in a ratio of 18.8:3.6:2.8:1.8:1.0.
