# Supplementary material for: Colonic bacterial composition is sex-specific in aged CD-1 mice fed diets varying in fat quality
Source: PLoS One. 2019 Dec 18;14(12):e0226635. doi: 10.1371/journal.pone.0226635 (PMC6919604; doi:10.1371/journal.pone.0226635)
Supplement: S2 Fig — (PDF) [file pone.0226635.s008.pdf]

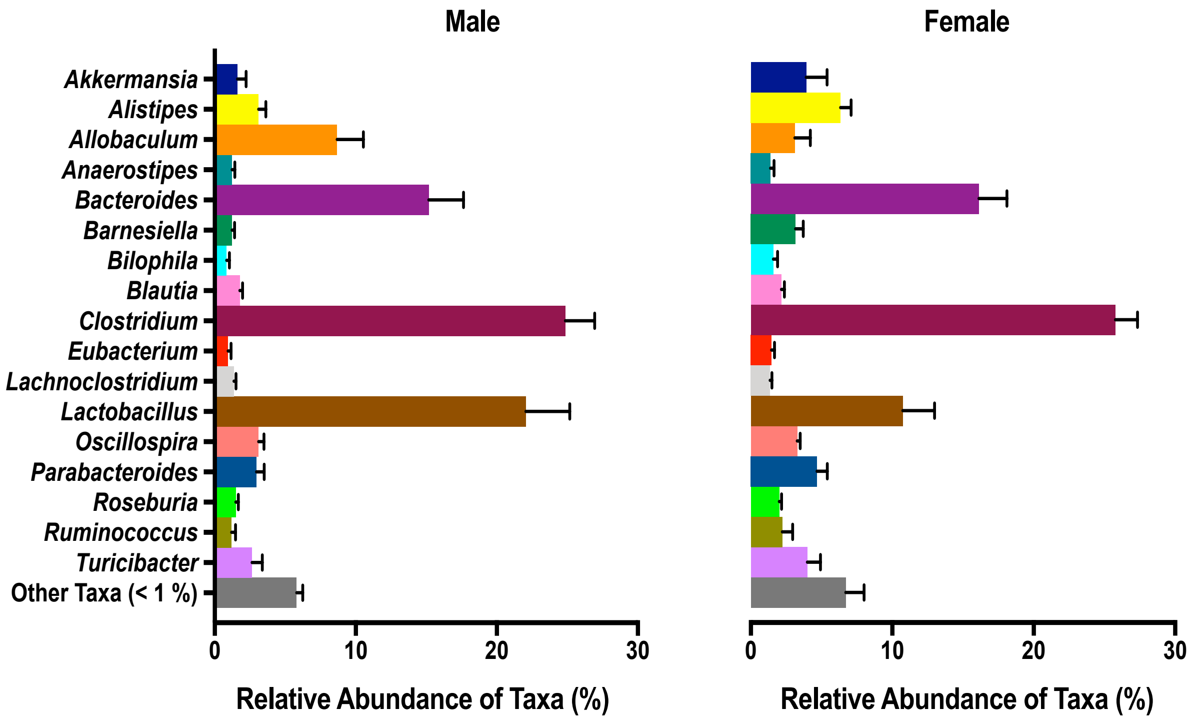

S2 Fig. Relative abundance of bacteria at the genus level of male and female CD-1 mice collapsed by diet group and age. Colonic abundance by counts of *Roseburia* was more abundant in females than males.
