# Supplementary material for: Colonic bacterial composition is sex-specific in aged CD-1 mice fed diets varying in fat quality
Source: PLoS One. 2019 Dec 18;14(12):e0226635. doi: 10.1371/journal.pone.0226635 (PMC6919604; doi:10.1371/journal.pone.0226635)
Supplement: S3 Fig — (A) Relative abundance of bacteria at the genus level of male and female CD-1 mice fed “Western-style” control fat (CO) at 10.5 and 13.5 months of age. (B) Relative abundance of bacteria at the genus level of male and female CD-1 mice fed CO supplemented with 30% fish oil (FO) at 10.5 and 13.5 months of age. (C) Relative abundance of bacteria at the genus level of male and female CD-1 mice fed CO supplemented with 30% dairy fat (BO) at 10.5 and 13.5 months of age. (D) Relative abundance of bacteria at the genus level of male and female CD-1 mice fed CO supplemented with 30% echium oil (EO) at 10.5 and 13.5 months of age. (PDF) [file pone.0226635.s009.pdf]

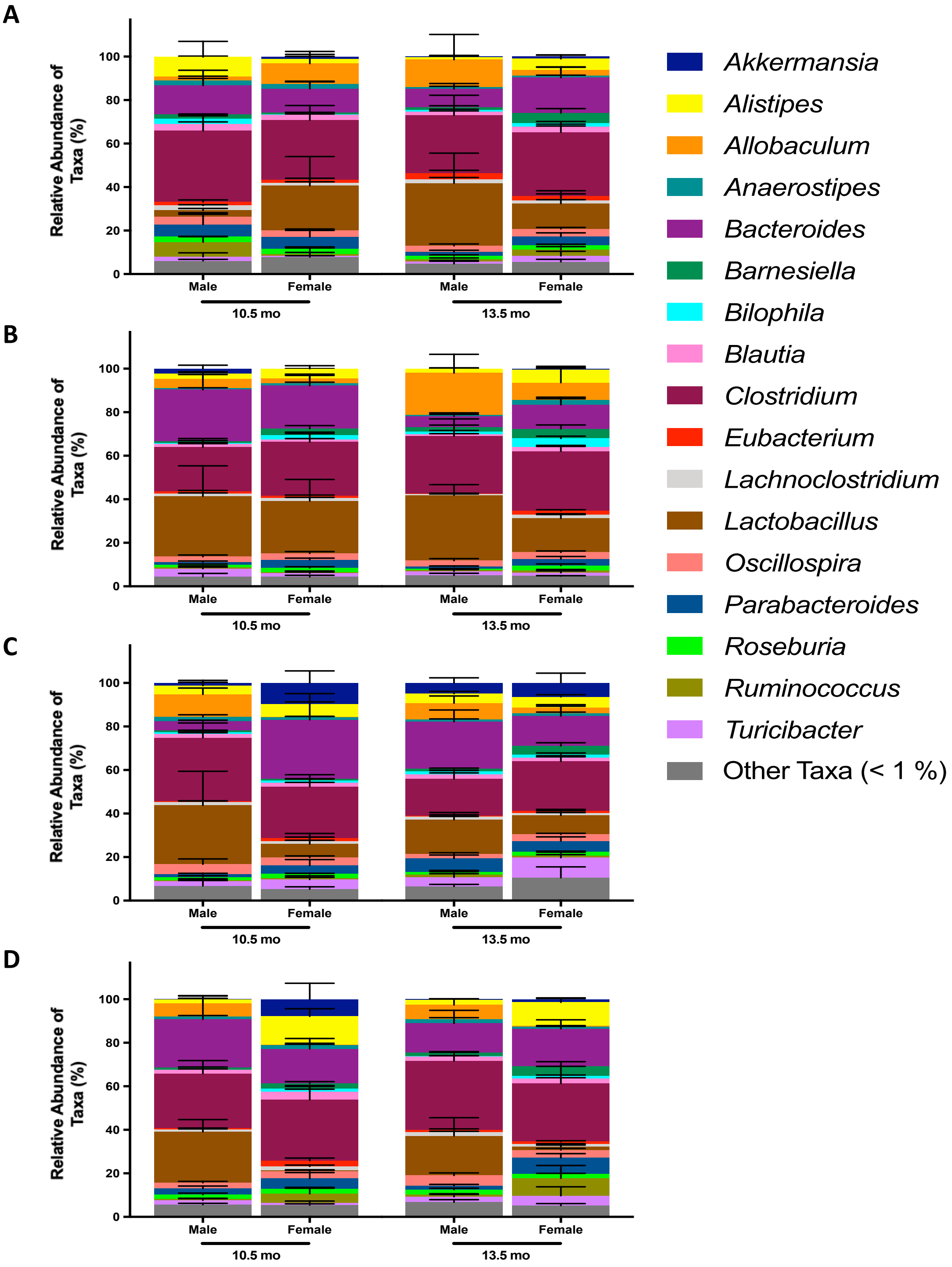

S3 Fig. (A) Relative abundance of bacteria at the genus level of male and female CD-1 mice fed "Western-style" control fat (CO) at 10.5 and 13.5 months of age. In males, the abundance by counts of *Alistipes* was lower at 13.5 months of age compared to 10.5 months of age ($P < 0.01$), but in females, the abundance by counts of *Alistipes* was greater at 13.5 months of age compared to 10.5 months of age ($P < 0.01$). **(B)** Relative abundance of bacteria at the genus level of male and female CD-1 mice fed CO supplemented with 30% fish oil (FO) at 10.5 and 13.5 months of age. In females, the abundance by counts of *Alistipes* was greater at 13.5 months of age compared to 10.5 months of age ($P < 0.05$). **(C)** Relative abundance of bacteria at the genus level of male and female CD-1 mice fed CO supplemented with 30% dairy fat (BO) at 10.5 and 13.5 months of age. In males, abundance by counts of *Bacteroides* was greater at 13.5 months of age compared to 10.5 months of age ($P < 0.05$). **(D)** Relative abundance of bacteria at the genus level of male and female

CD-1 mice fed CO supplemented with 30% echium oil (EO) at 10.5 and 13.5 months of age. In males, the abundance by counts of *Bacteroides* was lower at 13.5 months of age compared to 10.5 months of age ($P < 0.05$). Values are expressed as mean ± standard error of the mean.
