# Supplementary material for: Colonic bacterial composition is sex-specific in aged CD-1 mice fed diets varying in fat quality
Source: PLoS One. 2019 Dec 18;14(12):e0226635. doi: 10.1371/journal.pone.0226635 (PMC6919604; doi:10.1371/journal.pone.0226635)
Supplement: S4 Fig — (PDF) [file pone.0226635.s010.pdf]

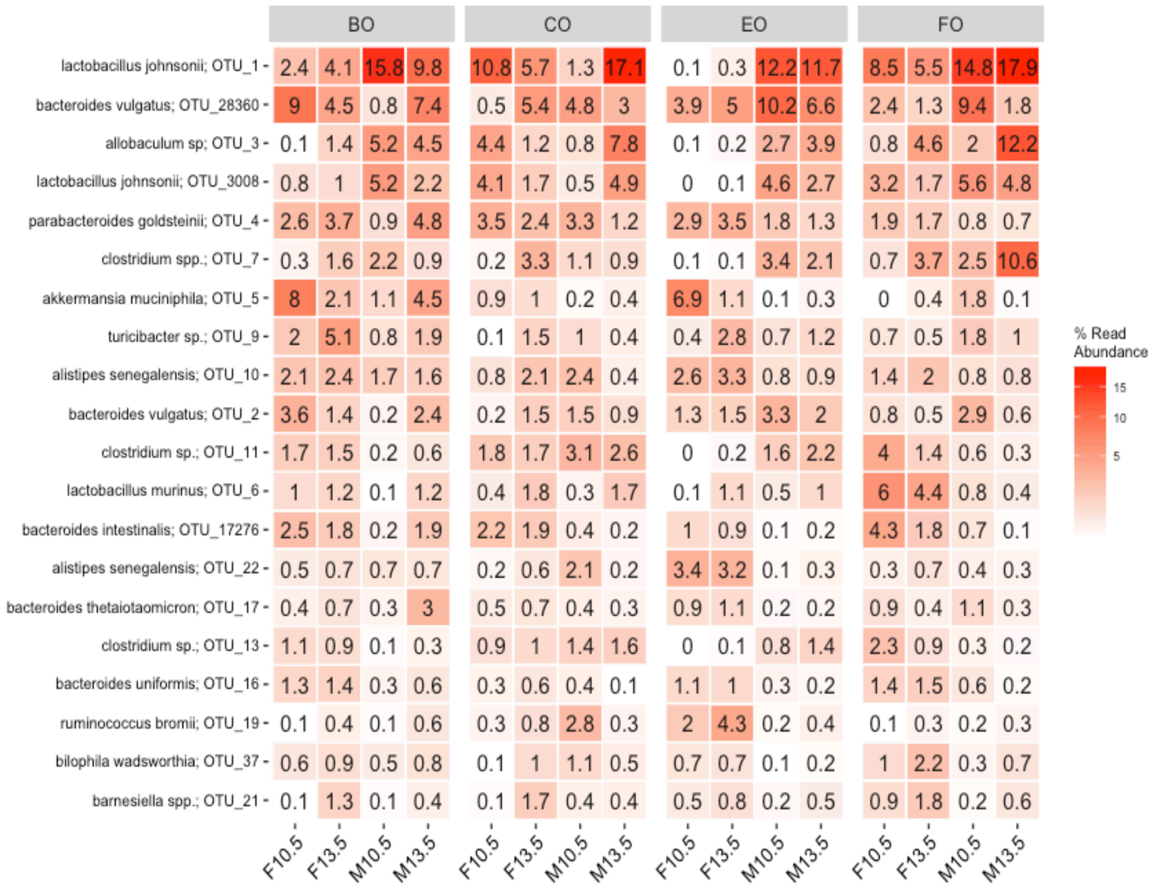

S4 Fig. Heat map of read abundance of the 20 most abundant colonic bacterial OTUs in CD-1 mice separated by diet, sex, and age. BO: Male (M) and female (F) CD-1 mice fed "Western-style" control fat (CO) supplemented with 30% dairy fat (BO) at 10.5 and 13.5 months of age. CO: Male (M) and female (F) CD-1 mice fed "Western-style" control fat (CO) at 10.5 and 13.5 months of age. EO: Male (M) and female (F) CD-1 mice fed CO supplemented with 30% echium oil (EO) at 10.5 and 13.5 months of age. FO: Male (M) and female (F) CD-1 mice fed CO supplemented with 30% fish oil (FO) at 10.5 and 13.5 months of age.
